# Single‐cell insights into the role of T cells in B‐cell malignancies

**DOI:** 10.1002/1873-3468.70099

**Published:** 2025-06-26

**Authors:** Laura Llaó‐Cid

**Affiliations:** ^1^ Institut d'Investigacions Biomèdiques August Pi i Sunyer (IDIBAPS) Barcelona Spain

**Keywords:** B‐NHL, immunotherapy, single‐cell analyses, T‐cell heterogeneity, tumor microenvironment

## Abstract

The intricate interplay between T cells and tumor cells in B‐cell malignancies has garnered significant attention in recent years. Single‐cell technologies have revolutionized our understanding of this dynamic relationship, offering unprecedented resolution and insights into cellular heterogeneity, signaling pathways, and immune interactions. This review synthesizes the recent findings that single‐cell methodologies have elucidated on the roles of T cells in the pathogenesis, progression, and therapeutic response of B‐cell malignancies. Key discoveries include the identification of previously unknown T‐cell subsets and their functional states, as well as the mechanisms of immune evasion by malignant B cells. These insights pave the way for innovative therapeutic strategies aimed at harnessing the immune system to combat B‐cell lymphomas.

## Graphical abstract

Single‐cell technologies have transformed our understanding of T cell–tumor cell interactions in B‐cell malignancies, revealing new T‐cell subsets, functional states, and immune evasion mechanisms. This Review synthesizes these findings, highlighting the roles of T cells in pathogenesis, progression, and therapy response, and underscoring their potential to support innovative immunotherapies for B‐cell lymphomas.

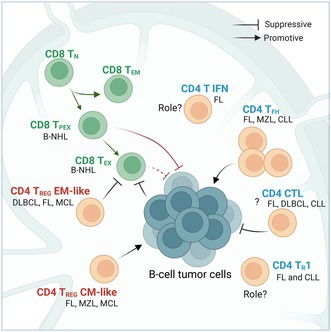

## Abbreviations

**AI**, artificial intelligence

**BCR**, B‐cell receptor

**BM**, bone marrow

**B‐NHL**, B‐cell non‐Hodgkin lymphoma

**BTK**, Bruton tyrosine kinase

**CAR‐T**, Chimeric antigen receptor‐T cell

**CD4 CTL**, cytotoxic CD4 T cells

**CD8 CTL**, cytotoxic CD8 T cells

**cDCs**, conventional dendritic cells

**CITE‐seq**, cellular indexing of transcriptomes and epitopes by sequencing

**CLL**, chronic lymphocytic leukemia

**CODEX**, spatial imaging with Co‐detection by indexing

**CRS**, cytokine release syndrome

**CyTOF**, cytometry by time of flight

**DCs**, dendritic cells

**DEGs**, differentially expressed genes

**DLBCL**, diffuse large B‐cell lymphoma

**FACS**, fluorescence‐activated cell sorting

**FL**, follicular lymphoma

**IC**, immune checkpoint

**ICANS**, immune effector cell‐associated neurotoxicity syndrome

**IFI6**, interferon alpha‐inducible protein 6

**IFIT1**, interferon‐induced protein with tetratricopeptide repeats 1

**IP**, infusion product

**ITK**, kinase tyrosine‐protein kinase ITK/TSK

**LN**, lymph node

**MBL**, monoclonal B‐cell lymphocytosis

**MCL**, Mantle cell lymphoma

**MHC**, major histocompatibility complex

**MZL**, marginal zone lymphoma

**NK**, natural killer cells

**PB**, peripheral blood

**pDCs**, plasmacytoid dendritic cells

**PSI**, polyfunctional strength index

**rLNs**, reactive LNs

**RNA‐seq**, RNA sequencing

**RS**, Richter syndrome

**scRNA‐seq**, single‐cell RNA sequencing

**SLECs**, short‐lived effector cells

**SLOs**, secondary lymphoid organs

**T**_**CM**_, central memory T cells

**TCR**, T‐cell receptor

**T**_**EF**_, effector CD8 T cells

**T**_**EM**_, effector memory T cells

**T**_**EMRA**_, effector memory re‐expressing CD45RA

**T**_**EX**_, exhausted T cells

**TF**, transcription factors

**T**_**FH**_, follicular helper T cells

**T**_**H**_, CD4 T helper cells

**TME**, tumor microenvironment

**T**_**MEM**_, memory T cells

**T**_**N**_, naive T cells

**T**_**PEX**_, precursor exhausted T cells

**T**_**R**_ **1**, regulatory 1 T cells

**T**_**REG**_, regulatory T cells

**T**_**TOX**_, cytotoxic T cells

B‐cell non‐Hodgkin lymphomas (B‐NHLs) represent a heterogeneous group of indolent and aggressive malignancies, including diffuse large B‐cell lymphoma (DLBCL), follicular lymphoma (FL), chronic lymphocytic leukemia (CLL), mantle cell lymphoma (MCL), and marginal zone lymphoma (MZL). They arise from B lymphocytes at various stages of development, and the characteristics of the specific lymphoma subtype reflect those of the cell from which they originated [1]. B‐NHL entities shape their T‐cell microenvironment in distinct manners, and this heterogeneity and its implications are only now starting to be understood. Over the past few decades, techniques such as fluorescence‐activated cell sorting (FACS) and bulk RNA sequencing (RNA‐seq) have significantly expanded our understanding of T‐cell phenotypes and their diverse functions in B‐cell neoplasms. It is now well established that T cells can exhibit opposing roles—either promoting or inhibiting tumor progression—depending on the specific type of T cell involved. For instance, regulatory T cells (Treg) and follicular helper T cells (Tfh) often display pro‐tumoral activities, contributing to an immunosuppressive environment and favoring tumor cell proliferation, respectively [2, 3]. In contrast, cytotoxic CD8 T cells (CD8 CTLs) typically exert anti‐tumoral effects by directly killing malignant B cells [4, 5].

Given the complexity and parallel functions of T cells, it is crucial to analyze them at a more granular level, considering both the specific subset and the activation or differentiation state of the cells. This detailed analysis is essential for unraveling the context‐dependent roles of T‐cell subsets within the tumor microenvironment (TME), which could provide insights into more targeted and effective immunotherapies. Understanding these nuanced interactions may help in developing strategies to exploit the anti‐tumoral potential of certain T‐cell populations while mitigating the pro‐tumoral effects of others.

**Fig. 1:**
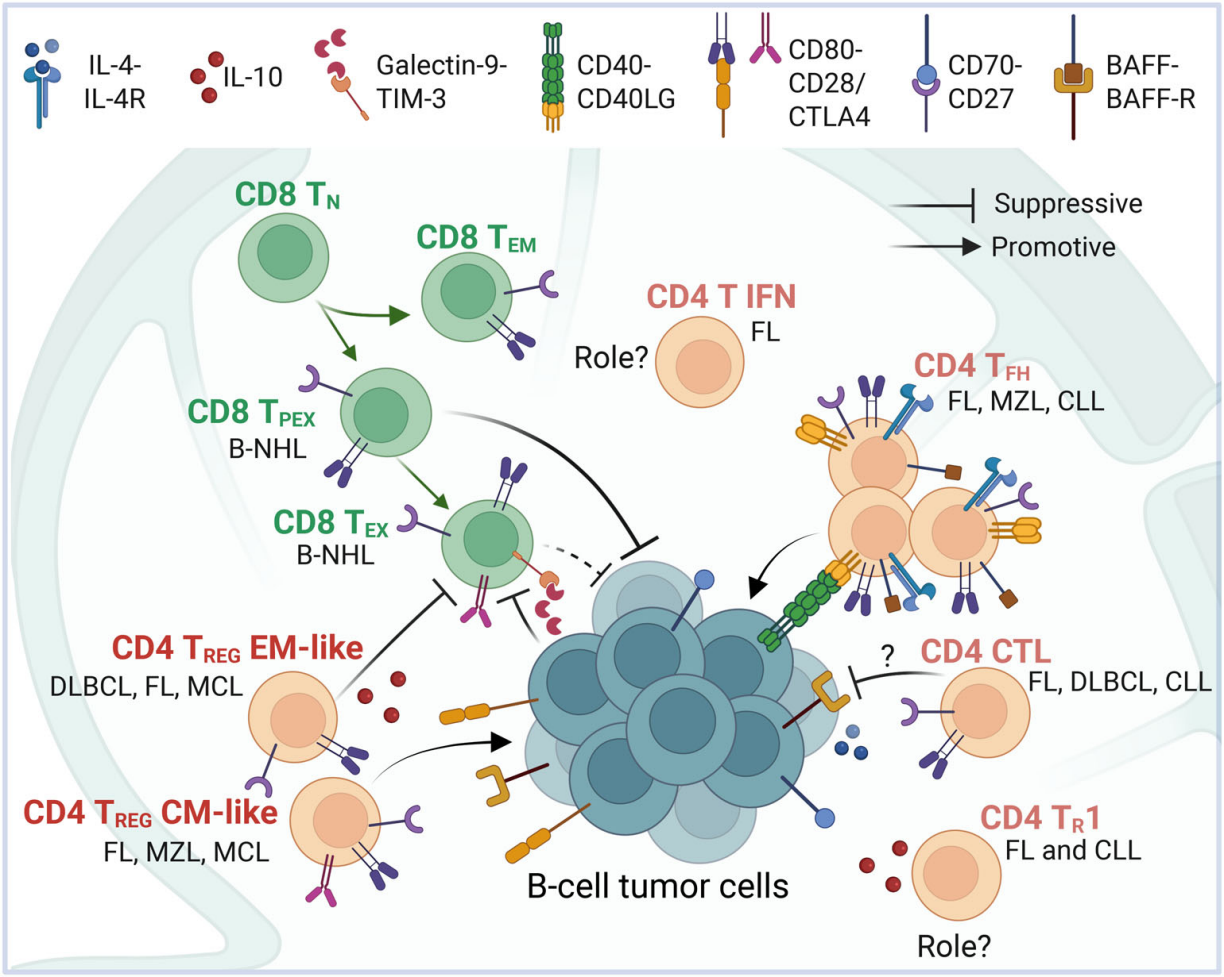
T‐cell subsets identified by single‐cell techniques in B‐cell malignancies. T‐cell subsets described using single‐cell studies. The differentiation pathway described for CD8 T cells is depicted. Key cell–cell interactions established between T cells and tumor cells are indicated. Potential roles in the tumor microenvironment of B‐NHL are depicted: black arrows indicate a promotion or positive influence to tumor survival or proliferation, whereas inhibitory lines represent suppressive effects to tumor or CD8 T cells. Created in BioRender. Llaó‐Cid, L. (2025) Created in BioRender. https://BioRender.com/xql7vv4.

With the advent of advanced single‐cell technologies—such as single‐cell RNA sequencing (scRNA‐seq), cellular indexing of transcriptomes and epitopes by sequencing (CITE‐seq), (imaging) mass cytometry, and spectral flow cytometry—we can now delve even deeper into the complexities of T‐cell biology within B‐cell neoplasms. The exact composition, quantity, and phenotypic diversity of T cells present in B‐NHLs can be accurately determined by analyzing individual cells. This new level of resolution allows us to construct a comprehensive T‐cell atlas in an unbiased manner, enabling the discovery of previously unknown T‐cell subsets that play pivotal roles in disease progression (Fig. 1). Moreover, the integration of spatial single‐cell technologies offers the opportunity to map how these cells are spatially organized within the tissue and how they interact with other components of the TME, allowing us to define alterations in tissue architecture and their implications for lymphoma evolution.

Altogether, this knowledge will be instrumental in identifying the key drivers of clinical response and discovering new therapeutic targets for patients with B‐NHL. This review highlights how single‐cell technologies have advanced our understanding of T cells in B‐cell malignancies, shedding light on their functional diversity, spatial organization, and potential as targets for novel therapeutic strategies.

## An atlas of T‐cell subsets and functional states in B‐cell lymphomas

Following antigen recognition and co‐stimulation, naive CD8 T (T_N_) cells are activated, undergo clonal expansion, and give rise to effector CD8 T (Tef) cells. Most of these CD8 T cells differentiate into short‐lived effector cells (SLECs), which die after antigen clearance. However, a small subset of this activated, clonally expanded pool differentiates into long‐lived memory CD8 T (T_MEM_) cells [6]. Until recently, memory T‐cell populations were considered to comprise central memory T cells (T_CM_), effector memory T cells (T_EM_), and effector memory T cells re‐expressing CD45RA [the long isoform of receptor type tyrosine‐protein phosphatase C (CD45)] (T_EMRA_). Traditional analyses have differentiated these cell subsets with surface protein markers including CD45RA, CD45RO (the short isoform of CD45), C‐C chemokine receptor type 7 (CCR7), and L‐selectin (CD62L). Recently, high‐dimensional technologies, such as cytometry by time of flight (CyTOF) and scRNA‐seq, have revealed a higher degree of diversity within memory T‐cell subsets, also in B‐NHL (Table 1). Studies measuring protein expression, such as CyTOF, typically use the traditional nomenclature and the aforementioned markers to distinguish T‐cell subsets. By contrast, scRNA‐seq studies cluster cells based on differentially expressed genes (DEGs) for manual or automated annotation, and, notably, these DEGs often differ from canonical markers. For example, CD45RA and CD45RO are isoforms of the *PTPRC* gene and cannot be well profiled by most scRNA‐seq technologies due to the difficulty of these methods in mapping short reads to genes. In addition, study design and analysis methodology influence the detection and definition of T‐cell populations. Consequently, there is considerable heterogeneity in nomenclature, with similar cell subsets named differently across studies.

**Table 1:** Studies that analyzed T cells in B‐NHL using single‐cell technologies.

References	Samples used in single‐cell analyses	Tumor type	Single‐cell technique	Cell populations identified	Therapy
Roussel *et al*. (2020) [7]	22 biopsies	7 DLBCL, 2 FL, 7 HL, and 6 HD	Mass Cytometry (32 markers)	CD4 T_N_, CD4 T_CM_, CD4 T_EM_, CD4 T_EMRA_, T_REG_, CD8 T_N_, CD8 T_CM_, CD8 T_EM_, and CD8 T_EMRA_	No
Ye *et al*. (2022) [8]	20 LN biopsies	17 DLBCL, 1 BL, 1 FL, 1 rLN	scRNA‐seq	T_N_, CTLs, T_REG_, T_FH_, and T_H17_	No
Liu *et al*. (2023) [9]	8 PCNS biopsies	8 DLBCL	scRNA‐seq	CD8 T_EX_‐1 to 6, CD8 prolif, CD8 T_MEM_‐like, CD4 T_REG_, CD4 T‐activated, CD4‐cytotoxic	No
Steen *et al*. (2021) [10]	8 LN biopsies	4 DLBCL, 3 FL, and 1 tonsilitis	scRNA‐seq	CD4 T cells S1‐S4, CD8 T cells S1‐S4, T_FH_ S1‐S3, T_REG_ S1‐S5	No
Andor *et al*. (2019) [11]	6 LN biopsies from patients, 3 PBMCs and 2 tonsils from healthy individuals	6 FL, 5 healthy individuals	scRNA‐seq	CD8 T_MEM_, CD4 T_REG_, CD4 T_MEM_, CD8 T_N_, CD4 T_N_	No
Haebe *et al*. (2021) [12]	20 LN biopsies	10 FL	scRNA‐seq	T‐activated, T_EX_, Tprolif, T_FH_ IL4, T_FH_, T_R_1, T_REG_, CD4 T_MEM_, T_IFN_, CD4 T_N_, CD8 T_N_, T_EF/MEM_	No
Wang *et al*. (2022) [13]	LNs of 73 FL patients and 34 normal or reactive LNs	73 FL and 34 tumor‐free	CyTOF (40 markers) and scRNA‐seq (of 6 FL and 4 rLN)	CD4 T_N_, CD4 T_CM_, CD4 T_EMRA_, CD4 T_REG_, CD4 TEM, T_FH_, T_H1_, CD8 T_CM_, CD8 T_N_, CD8 T_EM_, CD8 T_EMRA_	No
Han *et al*. (2022) [14]	23 LN samples	20 FL and 3 tumor‐free individuals	scRNA‐seq	CD8 T_N_, CD8 T_EX_, CD8 T_EF_, CD4 CTL, CD4 T_FH_, CD4 T_REG_, CD4 T_N_	No
Yang *et al*. (2023) [15]	82 biopsies from FL patients and 4 tonsils	82 FL and 4 tumor‐free	CyTOF (82 biopsies), CITE‐seq (4 FL LNs and 4 tonsils), and multiplex imaging mass cytometry (MxIMC) with Hyperion (3 FL LNs)	CD4^−^ CD8^−^ T cells, CD8 T_EMRA_, CD4 T_MEM_, CD4 T_N_, CD4 T_CM_, CD57^+^ SLEC, T_N_ MPEC, CD4 CD57^+^ T_FH_, CD4 CD57^+^ T_EX_, CD8 MPEC, CD8 CD26^+^ SLEC, CD4 CD57^+^ T_REG_, CD8 CD57^+^ SLEC, CD8 T_EX_, CD4 T_EX_, CD4 CD57^−^ T_REG_, CD4 PD1^+^ T_REG_, CD4 CD57^−^ T_FH_	No
Sarkozy *et al*. (2024) [16]	35 biopsies	FL (11 non‐transformed FL, 11 transformed FL‐FL, 11 transformed FL‐DLBCL) and 2 rLNs	scRNA‐seq and multi‐color IF	T_FH_ CD69, T_FR_, T_FH_, T_FH_ GITR, Tprolif, T_REG1_, T_REG_, T_EF_, T_EF_ CD27 LAG3, T_EF_ CYTO, T_CM_/T_N_, T_CM_/T_N_, T_CM_, T_IFN_, T_MEM_ CD69, NK/ NKT	No
Anagnostou *et al*. (2023) [17]	36 splenic bioipsies	36 MZL	CyTOF, CITE‐seq, and multiplex imaging mass cytometry (MxIMC) with Hyperion	CD8 T_N_, CD8 T_CM_, CD8 T_EF_, CD8 CD28^−^ SLEC, CD8 T_EMRA_ CD57^+^, CD8 T_EX_ CD57^+^, CD8 T_EX_ KLRG1^−^, CD8 MPEC, CD8 T_EX_ KLRG1^+^, CD8 T_EM_ KLRG1^+^, T_REG_ TIGIT^+^, T_REG_ TIGIT^−^, CD4 T_H1_, CD4 T_H2_, CD4 T_H17_, CD4 T_H22_, and CD4 T_FH_ ICOS^+^	No
Roider *et al*. (2020) [18]	12 LN biopsies	5 DLBCL, 4 FL, and 3 rLNs	scRNA‐seq	CD4 T_H_, CD8 T_TOX_, CD4 T_FH_, and T_REG_	No
Spasevska *et al*. (2023) [19]	59 LN tumor biopsies and 14 PBMC samples	29 FL, 17 DLBCL, 10 MCL, 12 HD, and 5 HD	CITE‐seq and TCR‐seq, mass Cytometry (33 markers), and Imaging mass Cytometry (25 markers)	Activated T_REG_, Resting T_REG_, LAG3^+^ T_REG_, CD4 T_N_, CD4 T_MEM_1‐3, CD4 T_FH_1‐2, CD4 T_H_, CD4 CTL, and CD4 Tprolif	No
Roider *et al*. (2024) [20]	51 LN biopsies	12 DLBCL, 8 MCL, 12 FL, 11 MZL and 8 HD	CITE‐seq, TCR‐seq, and spatial imaging by co‐detection by indexing (CODEX)	Tprolif, CD4 T_N_, CD4 T_H_ CM1‐2, CD4 T_FH_, T_REG_ CM1‐2. T_REG_ EM1‐2, CD8 T_N_, T_EM_1‐3. T_DN_	No
Radtke *et al*. (2024) [21]	39 LN biopsies	39 FL (16 Non‐Progressors, 18 Early Progressors, 10 Early Relapsers) and 5 tumor‐free individuals	Iterative bleaching extends mutiplexity (IBEX) inaging (39 antibodies) and scRNA‐seq	Tprolif, CD4 T_N_, T_FH_, T_REG_, CD8 T_N_, CD8 T_EX_, CD8 CTL	NA
Bachireddy *et al*. (2021) [22]	1 BM biopsy	1CLL (3 time‐points: pre‐treatment, remission and relapse)	scRNA‐seq (inDrop‐seq)	CD8 T_EX_‐like and CD8 T_PEX_‐like	Donor lymphocyte infusion (DLI)
Teh *et al*. (2023) [23]	38 PB samples of pre‐ and post‐treatment	19 CLL	CyTOF (39 markers)	CD4 T_N_, CD4 T_EM_, CD4 T_CM_, CD4 T_EMRA_, CD8 T_N_, CD8 T_EM_, CD8 T_CM_, and CD8 T_EMRA_	Venetoclax
Parry *et al*. (2023) [24]	17 BM samples	6 RT (4 Responders, 2 Non‐responders) and 2 CLL	CITE‐seq and TCR‐seq	CD8 T_EX_, CD8 T_EF/EM_, CD8 T_EF/EM_2, CD8 T_MEM_ GZMK^+^, CD4 CTL, T_REG_, CD4 T_N_, CD4.1, CD4.2	Ibrutinib +α PD‐1
Zhang *et al*. (2021) [25]	21 LNs from MCL patients and PBMCs from 2 healthy donors	5 MCL (3 Ibrutinib‐responsive and 2 nonresponsive) and 2 healthy individuals	scRNA‐seq	CD4 T, CD8 T	Ibrutinib
Rendeiro *et al*. (2020) [26]	12 PBMCs from different time‐points	4 CLL	scRNA‐seq	CD4 T, CD8 T	Ibrutinib
Purroy *et al*. (2021) [27]	20 PBMCs samples from two different time‐points	3 high‐count MBL patients, 7 CLL patients plus 2 CLL patients before and after Ibrutinib treatment, and 2 HD	scRNA‐seq	gdT, MAIT, CD8 T_N_, CD8 T_CM_, CD8 T_EM_, CD4 T_N_, CD4 T_CM_, CD4 T_EM_, CD4 CTL, T_REG_	Ibrutinib
Stéphan *et al*. (2024) [28]	18 PBMC samples post‐treatment	18 CLL (10 Ibrutinib‐responsive, 8 nonresponsive)	Spectral flow cytometry	20 CD8 T‐cell clusters (comprising T_N_, T proliferating and T_EF_), 21 CD4 T conventional clusters (comprising T_N_, T_H1_‐like, T_FH_ and GZMB+ CTL‐like), and 4 T_REG_ clusters (comprising T_N_‐like and T proliferating)	Ibrutinib
Wang *et al*. (2023) [29]	6 PBMC samples	6 DLBCL (3 durable response and 3 resistant patients)	scRNA‐seq	CD4 T_REG__T_H2__T_H17_, CD8 T_MEM_, CD8 T_EF_/NK‐like, CD8 T_N_/T_SCM_, CD8 Tprolif, Tprolif CD8 T_EMRA_, CD8 Cytotoxic, CD4 T_MEM_/T_H1_, Prolif CD8 T_EF_, CD4 T_REG_/T_EMRA_, CD8 T_EM_/Cytotoxic, CD8 T, CD8 T_EM_, T_H1_‐like/ T_EMRA_, CD8 T activation, CD8 T_EF_, T mix cytokine, Tprolif CD8 T_EMRA_, Mix T_REG_, CD8 Tprolif	CAR‐T
Zhao *et al*. (2023) [30]	PB samples before and after CAR‐T cell infusion	5 DLBCL (2 with non‐complete response, 3 with complete response)	scRNA‐seq	CD8 T_N_, CD8 T_EM_, CD8 Tprolif, CD8 T_EF_, CD8 Activated, CD4 T_N_, CD4 T_REG_	CAR‐T
Haradhvala *et al*. (2022) [31]	32 PBMC samples	32 DLBCL (treated with axi‐cel (*n* = 19) or tisa‐cel (*n* = 13))	scRNA‐seq	CD8 T_CM_, T_EM_, SLEC; CD4 T_EM_ CTL, T_REG_	CAR‐T
Louie *et al*. (2023) [32]	20 PBMC samples at different time‐points and CAR‐T products	2 DLBCL and 6 B‐ALL	scRNSA‐seq, protein (Ab‐seq), and mass cytometry (27 markers)	CD8 T cells: Cytotoxic – NK‐like, GNLY^+^, Cytotoxic – NK‐like, GNLY^−^, T_EM1_, T_EM2_, Activated‐prolif, T_EF_ MAIT, T_N/CM_	CAR‐T
Strati *et al*. (2023) [33]	14 BM samples	14 DLBCL (9 with persistent cytopenia, 5 without)	scRNA‐seq	CXCR1hi T_EF_, CXCR1lo T_EF_, KLRC2^+^ T_EF_, PPBP^+^ T_EF_, CTLA4^+^ T_EF_, S100A8^+^ T_EF_, MALAT1hi T_EF_, MAIT, CXCR4hi T_EF_, ISG^+^ T_EF_, T_N_	CAR‐T
Good *et al*. (2022) [34]	31 samples of CAR‐T products at day 7	31 DLBCL	CyTOF (34 markers) and CITE‐seq	10 metaclusters by CyTOF; CD4 CD57^+^ Tbet^+^, CD4 CD57^−^ Helios^+^, CD8 CD57^+^ T‐bet^+^ by scRNA‐seq	CAR‐T
Deng *et al*. (2020) [35]	24 CAR‐T infusion products	16 DLBCL, 6 tFL, 2 PMBCL	scRNA‐seq	CD4 T_MEM_, CD8 T prolif, CD4 T prolif, CD8 T prolif, CD8 T_EX_, T_H17_, GATA3^+^ CD8, CD7^+^ CD8, CD8 T_EM_, CD8 T_EF_ (activated), T_REG_, CD8 T‐cells, Activated CD4, CD4 T cells, CD8 T prolif, CD8 T (LAMP high), KLRC1^+^, IACs	CAR‐T
Jin *et al*. (2024) [36]	20 biopsies	13 rrDLBCL	Imaging mass cytometry (31 markers)	CD4 T_N_, CD4 T_CM_, CD4 T_EM_, CD4 T_EF_, T_REG_, T_H1_, T_H2_, CD8 T_N_, CD8 T_CM_, CD8 T_EM_, CD8 T_EF_	CAR‐T
Jiang *et al*. (2022) [37]	39 samples of PB, BM, apharesis or biopsises	15 MCL	scRNA‐seq	CD8 T_EX_, CD8 T_MEM_, CD4 T_MEM_, T_REG_, T_N_, T prolif, CD8 CTL, CD4 CTL, gdT	CAR‐T
Melenhorst *et al*. (2022) [38]	10 PB samples	2 CLL	CyTOF, and CITE‐seq	CyTOF: CD4 Ki67hi, CD4 T, CD8 GZMK, CD8 GZMB, gdT Helioshi; CITE‐seq: Non‐CAR T CD4, Non‐CAR T CD8, CAR T CD4, CAR T CD4 S phase, CAR T CD4 G2/M phase	CAR‐T
Derigs *et al*. (2024) [39]	7 samples of CAR‐T infusion products	7 CLL (4 responders and 3 non‐responders)	Spectral flow cytometry (36 markers)	CD8 T_N_, CD8 T_EF_ CD56^+^, CD8 T_CM_ CD39^+^, CD8 T_EM_, CD8 T_CM_ CD314^+^, CD8 T_EM_ CD314^+^, CD8 T_EM_ CD39^+^, CD8 T_EM_ CD197, CD8 T_EM_ CD197^+^ CD39^+^, CD4 T_CM_, CD4 T_EM_, CD4 Tn, CD4 T_EM_ CD39^+^ PD1^+^, CD4 T_EM_ PD1^+^, CD4 T_EM_ CD56^+^, CD4 T_EM_ CD197^+^	CAR‐T
Denlinger *et al*., 2024 [40]	26 post‐infusion PBMC samples	26 CAR‐T‐treated B‐NHL patients (16 complete‐responders and 10 with progressive disease). 16 DLBCL, 3 HGBCL, 2 PMBCL, 1 RT, 1 MCL, and 3FL	Spectral flow cytometry (37 markers)	14 clusters of CD8 CAR T cells, including clusters of PD1^+^ TCF1^+^ (Clusters 8 and 9), PD1^+^ TCF1low TOX^−^ (Clusters 7 and 13), PD1^+^ TIM‐3^+^ T‐bet^+^ GZMB^+^ (Clusters 11 and 12), and PD1^−^ T‐bet^+^ GZMB^+^ (Clusters 2, 3, and 4)	CAR‐T
Sheih *et al*. (2020) [41]	16 samples of CAR‐T product at different time‐points	2 NHL, and 2 CLL	scRNA‐seq	4 clusters of CD8 CAR‐T: activation‐, cytotoxicity‐, mitochondrial‐, and cell cycle‐associated genes	CAR‐T
Jackson *et al*. (2022) [42]	24 samples of CAR‐T cell products at different time‐points	15 NHL patients	scRNA‐seq and flow cytometry	CD4 T_N_, CD4 T_EF_, CD8 T_CM_, CD8 T_EM_, CD8 T_N_, CD8 T_EF_, T_FH_, T_H1_, T_H1_/T_H17_, T_H17_7, T_H2_, T_REG_	CAR‐T
Rossi *et al*. (2018) [43]	22 CAR‐T product samples	19 DLBCL, 2 FL and 1 MCL	Single‐cell multiplex cytokine profiling on the IPs product		CAR‐T
Maurer *et al*. (2024) [44]	77 PBMC samples (CAR‐T infusion products and at different time‐points)	28 DLBCL patients (15 responders and 13 non‐responders)	scRNA‐seq, CyTOF, and Multiparameter Flow Cytometry	CD4 CTL, CD4 T_CM_, CD4 T_EM_, CD8 T_N_, CD8 T_CM_, CD8 T_EM_, and CD4 T_REG_	CAR‐T

### Effector memory and exhausted CD8 T cells

An increase in antigen‐experienced CD8 T cells accompanied by a reduction in CD8 T_N_ frequency [7, 20, 45, 46, 47] has been observed in B‐NHL by conventional flow cytometry, and this has been corroborated in recent single‐cell studies [7, 20, 27, 46, 48, 49]. Additional functional studies have suggested that such an increase might be due to anti‐tumor T‐cell activity [4, 5]. The increase in T_EF_ and/or T_EM_ CD8 cells has been linked to better or worse outcomes depending on the disease entity. While higher frequencies of these cells in FL are associated with an improved outcome [50], the opposite is observed in DLBCL [51].

Upon chronic antigen exposure and reception of inhibitory signals, T_EM_ cells acquire an exhausted phenotype characterized by the increased expression of inhibitory receptors such as programmed cell death protein 1 (*PDCD1*; encoding PD‐1), hepatitis A virus cellular receptor 2 (*HAVCR2*; TIM‐3), lymphocyte activation gene 3 protein (*LAG3*), cytotoxic T‐lymphocyte protein 4 (*CTLA4*), and T‐cell immunoreceptor with Ig and ITIM domains (*TIGIT*), reduced cytotoxic capacity, and impaired proliferation potential [52, 53].

Single‐cell studies in chronic infections and solid tumors have shed additional light on the heterogeneity of this cell state: CD8 T cells follow a differentiation path originating from CD8 Tn cells that diverges into either CD8 T_EM_ cells or exhausted CD8 (T_EX_) cells [54, 55, 56]. The intermediate population that gives rise to terminally Tex cells has been defined as CD8 Tem precursor exhausted cells (T_PEX_) [57]. Transcriptionally, they express intermediate levels of inhibitory receptors such as *PDCD1* and *LAG3*, and high granzyme K (*GZMK*). Importantly, these cells are responsible for the proliferative burst that occurs in response to anti‐PD‐1 blockade therapy (αPD‐1), correlating with improved patient outcomes in melanoma and lung cancer [56, 58]. Early flow cytometry studies reported increased expression of several inhibitory receptors and a diminished cytotoxic capacity in T cells from individuals with B‐NHL compared to healthy controls [5, 59, 60, 61]. Subsequent scRNA‐seq and CyTOF analyses specifically identified an increased Tex population in tumors [7, 9, 22, 24, 61, 62, 63]. In addition, several of these studies describe cells with a similar transcriptional phenotype to T_PEX_ cells, with high expression of *GZMK* and at an earlier differentiation state compared to Tex cells, although few studies label them as such [9, 20, 22, 24]. Notably, the role of T_PEX_ in driving immune checkpoint blockade response has not yet been investigated in B‐cell neoplasias.

### CD4 T cells

Upon antigen exposure, CD4 T_N_ cells become activated and, depending on the surrounding stimulatory milieu and antigenic signals, differentiate into distinct CD4 helper T (T_H_) subsets, T_FH_ cells, or T_REG_ cells [62, 63]. Similar to the heterogeneity in naming for CD8 T cells, CD4 T cells are also differently defined depending on the technology used. Protein‐based studies often define CD4 T cells as CD4 T_N_, CD4 T_CM_, CD4 T_EM_, CD4 T_FH_, and CD4 T_REG_. In addition, CD4 Th cells can be further classified into T_H1_, T_H2_, T_H9_, T_H17_, and T_H22_ by the expression of transcription factors (TFs), cytokines, and surface markers. Largely due to data sparsity from dropout events that mask lowly expressed transcripts such as cytokines and TFs, most scRNA‐seq studies do not capture the T_H2_, T_H9_, and T_H22_ subsets [64, 65]. As a consequence, most studies classify CD4 T cells into CD4 T_N_, CD4 T_EM_, CD4 T_H_, CD4 T_H17_, CD4 CTL, CD4 T_FH_, and CD4 T_REG_ cells.

### CD4 T helper cells

CD4 T_H_ cells are responsible for modulating other immune cells by releasing cytokines [18, 66]. Traditional studies in solid tumors have shown that CD4 T_H_ cells sustain anti‐tumor activity by directing other leukocytes, enhancing tumor‐antigen presentation, and directly inhibiting tumor proliferation *via* interferon gamma (IFN‐γ) and tumor necrosis factor alpha (TNF‐α) [67]. Such functions, however, might not be equivalent in lymphomas, where tumor cells are antigen‐presenting cells that benefit from CD4 T_H_‐secreted cytokines. For example, it has been shown that INF‐γ secreted by T_H1_ cells activates CLL cells and induces their proliferation [68, 69]. Single‐cell studies have identified T_H_ subsets in B‐NHL, including T_H1_, T_H2_, T_H17_, and T_H22_ [8, 13, 17, 18, 19]. By CyTOF, the frequency of T_H1_ cells has been found to increase in FL compared to control lymph nodes (LNs) [13], whereas T_H17_ and T_H22_ are decreased in MZL [17]. Other protein and RNA‐based single‐cell studies that only discern T_H_ cells without specifying the subtype, or define CD4 T cells as T_MEM_ and T_CM_, describe an increase, a decrease, or no changes of these cells in B‐NHL [7, 18, 19, 20]. Notably, every work defines these subsets using different markers, making cross‐study comparisons difficult. Some studies have defined newly identified subsets: using scRNA‐seq in FL patients, Haebe *et al*. and Sarkozy *et al*. identified a cluster of T cells that respond to IFN signaling and exhibit high expression of genes such as interferon alpha‐inducible protein 6 (*IFI6*) and interferon‐induced protein with tetratricopeptide repeats 1 (*IFIT1*) [12, 16]. However, the role of this subset has not been explored in any of the studies. Of note, it is a small subset of cells, and its frequency seems to be decreased in FL LNs compared to cancer‐free reactive LNs (rLNs), which are LNs enlarged due to benign immune responses such as infection or inflammation [16].

### CD4 follicular helper T cells

T_FH_ cells are essential for germinal center formation and function, providing help signals to B cells to produce high‐affinity antibodies [70]. They can be identified by the expression of PD‐1, calcineurin B‐like protein 6 (CBL6), C‐X‐C chemokine receptor type 5 (CXCR5), and inducible T‐cell costimulator (ICOS) markers [71]. T_FH_ cells are enriched in LNs of FL, MZL, and CLL patients compared to rLNs [13, 17, 18, 20, 21, 72]. In solid tumors like breast and pancreatic cancer, *in vitro* and bulk RNA‐seq studies have linked T_FH_ cells to a positive outcome [73, 74]. However, recent scRNA‐seq analyses have associated them with worse outcomes in FL [10, 12, 13]. Studies including a large number of cells have revealed a certain degree of heterogeneity within T_FH_ cells [10, 15, 19]. For example, Sarkozy *et al*. [16] identified three subsets of T_FH_ – T_FH_, T_FH_ CD69, and T_FH_ GITR – that decrease upon FL transformation into DLBCL. Haebe *et al*. [12] described a subpopulation defined by high expression of *IL4* and *TNF* in FL, similar to a previously defined subset that supports malignant B cells *via* IL‐4 [75]. Last, using both CyTOF and scRNA‐seq analyses, Yang *et al*. [15] also identified a subset of CD57^+^ T_FH_ with a terminally differentiated phenotype correlating with inferior outcomes in FL patients.

### Cytotoxic CD4 T cells

CD4 T_EM_ cells with a cytotoxic profile (termed CD4 CTLs) expressing granzymes, perforin, and the TF eomesodermin (EOMES) were first identified by flow cytometry in murine melanoma tissue samples [76, 77] and in human solid tumors [78]. Single‐cell studies have since broadly detected them in solid tumors such as non‐small‐cell lung cancer, colorectal cancer, and breast cancer [79, 80, 81, 82]. They have been suggested to exert an anti‐tumor function, as they are clonally expanded in bladder tumor compared with adjacent normal tissue, and can directly kill autologous tumor cells *in vitro* [83].

In B‐NHL, CD4 CTLs were initially observed in CLL and DLBCL by flow cytometry [84, 85] and later confirmed in FL, DLBCL, and CLL *via* single‐cell studies [9, 14, 19, 24, 27, 37]. Combining both scRNA‐seq and T‐cell receptor sequencing, Spasevska *et al*. [19] demonstrated their clonal expansion compared to other CD4 T cells. Similarly, we found that clonally expanded CD4 CTLs are enriched in CLL LNs compared to rLNs [86]. However, their role in B‐NHL tumors remains to be explored.

### Regulatory CD4 T cells

T_REG_ cells are crucial for maintaining peripheral tolerance by preventing autoimmunity and chronic inflammation [87]. They are easily distinguishable by forkhead box protein P3 (FOXP3), CTLA4, and interleukin‐2 receptor subunit alpha (IL2RA) expression. Tregs have been widely described to be increased in B‐NHL [88, 89, 90], a finding further confirmed by single‐cell studies [13, 17, 18, 19]. Higher single‐cell sequencing/phenotyping depth has revealed two main T_REG_ subsets: activated T_REG_ (or T_EM_‐like) and resting T_REG_ (or T_CM_‐like) [19, 20]. Spasevska *et al*. [19] analyzed T_REG_ cells from LNs of DLBCL, FL, and MCL patients at the single‐cell protein and RNA level and found that activated Tregs were present at higher frequencies in tumor LNs and were associated with shorter progression‐free survival in FL. In addition, Roider *et al*. [20] found that CD69^+^ central memory T_REG_ (CD69^+^ T_REG_ CM2) cells were increased in FL and MZL, and zinc finger protein Aiolos (IKZF3^+^) effector memory Tregs (IKZF3^+^ T_REG_ EM) frequencies were increased in MZL, FL, and MCL compared to rLNs.

A scRNA‐seq analysis of 10 FL samples identified an additional T‐cell subset: regulatory 1 (T_R_1) cells, which express CTLA4, LAG3, and IL10 [12]. T_R_1 cells have been described in solid tumors using both traditional flow cytometry and scRNA‐seq techniques and are associated with disease progression [91, 92], whereas in a mouse model of CLL, it has been suggested that they control disease development [93].

### T‐cell phenotypes in B‐NHL versus normal lymphoid tissue

In healthy lymphoid tissues, CD8 and CD4 T cells sustain a balanced mix of naive, effector, and memory states, with minimal exhaustion and T_REG_ activity, to support immune responses [94]. Single‐cell studies have helped to reveal a stark shift in B‐NHL: CD8 T_N_ cells decrease as T_EX_ and T_PEX_‐like cells increase, likely driven by chronic antigen exposure and tumor immunosuppression [12, 14, 15, 17, 20, 21, 86]. Similarly, CD4 subsets such as T_FH_ and T_REG_ cells expand in entities such as FL and MZL, differing from their controlled roles in normal germinal centers [13, 17, 20]. Tissue‐specific niches further complicate this picture: LN, bone marrow (BM) and spleen harbor distinct microenvironments, influencing T‐cell phenotypes differently. Although some studies explore these compartments [17, 24], a comprehensive overview integrating their contributions is still missing.

## Different B‐cell lymphoma entities distinctly alter the tissue architecture, enriching specific cellular neighborhoods

Although most studies center their comparisons between disease entities and healthy controls, a few studies have compared different B‐NHLs to ascertain their commonalities and singularities. In this respect, Roider *et al*. [20] generated a resourceful dataset of 51 LN samples from DLBCL, MCL, FL, MZL, and rLNs, which were analyzed *via* CITE‐seq and spatial imaging with co‐detection by indexing (CODEX) technology. Tumor‐free rLNs were most distinct from malignant LNs, but, interestingly, the different lymphoma entities also substantially differed from each other. Specifically, DLBCL was enriched in terminally exhausted T cells [termed cytotoxic (T_TOX_) EM3 PD1^+^ TIM3^+^] and depleted of T_FH_ cells. Instead, MZL and FL were enriched with T_FH_ and a specific T_REG_ subset (IKZF3^+^ Treg EM2), whereas another T_REG_ subset (CD69^+^ T_REG_ CM2 cells) was increased in MCL, FL, and MZL. These entity‐specific T‐cell compositions resulted from differential clonal expansion of CD4 and CD8 T‐cell subsets. At a spatial level, B‐NHLs showed a disruption of the LN architecture and created unique microenvironmental patterns, preserving more or less structure. For example, DLBCL LNs, the least structured, exhibited a diffuse excess of a spatial neighborhood composed of T_EX_ cells and macrophages; FL LNs were re‐organized with an increased amount of germinal‐center‐like areas with T_FH_ and follicular dendritic cells and surrounded by areas containing T_REG_, memory T_H_ cells, T_EX_ cells, and malignant B cells. In contrast, MCL LNs presented little T‐cell infiltration, a significant predominance of follicle‐like B‐cell areas, and an absence of germinal centers. These findings reveal distinct T‐cell profiles—DLBCL's exhaustion dominance, FL and MZL's T_FH_‐T_REG_ enrichment, and MCL's T‐cell scarcity—driving entity‐specific immune evasion and microenvironmental adaptations, underscoring the need for such analyses to advance pathogenesis understanding and develop targeted treatment strategies.

In addition, the study of cellular ecosystems, beyond just analyzing individual cell subsets, has revealed their connections to disease molecular subtypes and clinical survival, providing novel opportunities for therapeutic targeting [10, 95]. For example, Steen *et al*. [10] developed the machine‐learning tool EcoTyper to analyze bulk and scRNA‐seq data and identified 44 cell states organized in nine cellular ecosystems in DLBCL. Among these, a CD8 T‐cell state (termed S1), resembling stem‐like CD8 T cells, was associated with improved therapeutic outcomes in patients receiving a combination of chemotherapy and bortezomib.

## Discerning between normal B–T cell interactions and malignant mechanisms of immune evasion *via* cell–cell communication analyses

In healthy secondary lymphoid organs (SLOs), such as the LN and the tonsil, B cells and T cells engage in tightly regulated interactions to generate immune responses and tolerance. B cells present antigens *via* major histocompatibility complex (MHC) molecules to CD4 T_FH_ cells, which, in turn, provide co‐stimulatory signals, like CD40‐CD40L as well as cytokines, such as IL‐4 and IL‐2, driving B‐cell activation, proliferation, and differentiation into plasma or memory B cells [96]. At the same time, T cells receive co‐stimulatory signals, including CD86‐CD28 and ICOS‐ICOSL, that promote activation and differentiation [70]. In their turn, CD4 T_REG_ cells modulate these responses to maintain homeostasis and inhibit immune cells through interactions such as CTLA4–CD80/CD86, and secretion of IL‐10 and TGFβ [97].

Recent scRNA‐seq analyses have provided deeper insights into these processes. For example, a comprehensive single‐cell atlas of the human tonsil [98] has mapped these interactions at unprecedented resolution, identifying key molecular circuits and cellular states, providing an important baseline for studying malignant alterations.

Coculture and RNA‐seq bulk studies have unraveled how communication between T cells and malignant B cells strongly influences tumor progression and therapy resistance in B‐NHL [99]. Exploiting ligand–receptor interaction databases, scRNA‐seq data now enables unbiased inference of the entire network of intercellular signaling among TME and tumor cells. Several tools have been developed for this objective, including CellPhoneDB [100], CellChat [101], and iTALK [102]. Using such analyses, several studies have identified the main interactions established between T‐cell subsets, malignant B cells, and other TME cells like monocytes and dendritic cells (DCs). Reminiscent of their role in healthy lymphoid tissues, many identified interactions between T cells and tumor B cells are common among B‐NHL. For example, the tumor‐supporting signals CD40–CD40L, BCMA–BAFF, and BAFF‐R–BAFF were described between CD4 T_H_ and T_REG_ cells and tumor B cells in DLBCL, CLL, and MCL [8, 18, 27]. Tumor B cells were found to stimulate both CD4 and CD8 T cells *via* CD86–CD28 and CD70–CD27 [8, 18, 25, 27, 86]. In addition, conventional DCs (cDCs) and plasmacytoid DCs (pDCs) interacted with CD4 T cells *via* ICOSLG–ICOS, and natural killer (NK) cells engaged T cells through TNF superfamily members and receptors [8]. IL4–IL4R and IL4–IL13RA1 interactions were exclusively observed between T_FH_ and malignant B cells [18]. Similar to their spatial singularities, a study identifying the unique interactions present in each tumor entity could provide valuable insights into the specific pathogenetic mechanisms driving tumorigenesis. Importantly, these are interactions inferred from transcript expression, and studies report moderate mRNA–protein concordance (40–70%), especially in dynamic phases such as cell differentiation [103, 104]. Thus, validations at protein and spatial levels should be added to confirm these observations and account for cell proximity and ligand–receptor co‐localization.

Although immune checkpoint blockade against PD‐1 or PD‐L1 has had high success in numerous solid cancers, including melanoma, renal cell cancer, and NSCLC, their effect in B‐NHL has been mostly restricted to DLBCL and FL with a modest effect [105]. Cell–cell interaction analyses have shed some light on the suppressive signals that tumor B cells and other immune cells such as T_REG_ and myeloid cells provide to T cells. Whereas the inhibitory interaction CD274–PDCD1 was only observed in DLBCL [8], interactions commonly present in most studies were CD80/CD86–CTLA4 and LGALS9–HAVCR2 [8, 18, 27, 37, 86]. In addition, NECTIN2/PVR–TIGIT was observed from myeloid cells, cancer‐associated fibroblasts, and endothelial cells to CD8 T cells in DLBCL [8], and BTLA/MIF–TNFRSF14 from tumor cells to T cells in MBL/CLL [27].

As already mentioned, many of these interactions are known to be occurring in nonmalignant SLOs between B cells and T cells. Thus, cell–cell communication analyses may include control samples and prioritize differential interactions across tumor and control LNs to identify tumor‐specific or tumor‐enriched interactions. By applying this differential analysis using CellChat, we identified HLA–LAG3, BTLA–CD247, ENTPD1–ADORA2A, and LGALS9–TIM3 ligand–receptor pairs as relevant inhibitory signals in CLL LNs, which were absent in rLN [86]. The identification of these different immune checkpoints suggests that they might play a more significant role than PD‐1 in B‐cell malignancies. In this line, some of these immune checkpoint (IC) inhibitors are currently being tested in clinical trials, including CTLA4 and LAG‐3 in combination with anti‐PD‐1, anti‐CD20, or BTK inhibitors [106, 107, 108].

By expressing adhesion molecules and secreting chemokines, lymphoid stromal cells promote malignant B‐cell survival and protect them from chemotherapy [109]. T cells are also known to interact with stromal cells [110]. For example, fibroblastic reticular cells in healthy SLOs secrete C‐C motif chemokine 19 (CCL19) and CCL21 to direct T cells into their T cell zone [111]. Despite the role of lymphoid stromal cells, they remain poorly characterized in both healthy and malignant states, with in‐depth single‐cell analyses only recently emerging [112]. Mourcin *et al*. [113] explored the phenotypic landscape of these cells and their polarization driven by interactions with tumor B cells in FL. The specific interplay between these stromal cells and T cells in B‐NHL remains to be further explored.

## Deciphering mechanisms of response for novel clinical agents with single‐cell technologies

Despite the extensive advances in managing and treating B‐NHL achieved with novel agents, including rituximab, ibrutinib, and chimeric antigen receptor (CAR‐T), challenges including resistance, relapse, and adverse events still exist. Thus, it is of paramount importance to identify molecular and cellular players responsible for therapeutic responses. Several single‐cell studies have provided new insights into the role of T cells in influencing treatment outcomes, mainly for ibrutinib and CAR‐T (Table 2).

**Table 2:** Role of T‐cell subsets upon treatment with novel clinical agents in B‐cell lymphomas.

Therapy	Category	T‐cell subset	Key findings and implications	References
Ibrutinib	Response	CD8 T_EF_	Higher proportion in responders' PB vs. non‐responders in MCL	[25]
	No response	CD4 and CD8 T cells	Increased LGALS1‐CXCR4/CD69, TGFB1‐CXCR4 in resistant patients	[25]
	Other	General T cells (post‐treatment)	Downregulation of inhibitory signals (e.g., BTLA/MIF‐TNFRSF14, CTLA4‐CD86)	[27]
			↓ leukocyte function, ↑ quiescence/senescence genes	[26]
PD‐1 blockade	Response	ZNF683^+^ CD8 T_EF/EM_	Clonally expanded, ↑ in RS responders	[24]
CAR‐T therapy	Response	Memory/stem‐like CAR‐T	↑ in responders' IP and PB (DLBCL, MCL, CLL)	[29, 31, 35, 40, 44]
		Effector CAR‐T	CD4/CD8 with senescence features linked to durable control in some studies	[34]
		Long‐term CD4 CAR‐T	Dominate in long‐term remission in CLL, proliferative/cytotoxic	[38]
	No response	Exhausted CAR‐T	↑ CD4 and CD8 T_EX_ in non‐responders	[29, 35, 39]
		CD4 T_REG_	↑ in non‐responders; but linked to less severe neurotoxicity	[31, 34]
	Adverse events	CX3CR1^+^ INF‐γ^+^ CD8 T cells	↑ in BM of patients with prolonged cytopenia	[33]
		IL‐17A‐polyfunctional (T_H17_)	↑ in severe ICANS	[43]
		Polyfunctional CAR‐T	↑ in responders and in severe CRS cases	[43]

### T‐cell implication in response to ibrutinib and anti‐PD‐1 treatments

#### Ibrutinib

Ibrutinib treatment has shown impressive efficacy in B‐NHL, although virtually all patients eventually relapse [114]. Thus, current research efforts focus on understanding mechanisms driving therapeutic resistance and identifying predictors of response. Ibrutinib inhibits the bruton tyrosine kinase (BTK), a downstream signaling molecule of the B‐cell receptor (BCR). BTK inhibition in malignant B cells not only inhibits proliferation and induces apoptosis but also prevents B cells from responding to survival stimuli provided by the TME [115]. In addition, it has been shown that ibrutinib inhibits the T‐cell receptor (TCR) downstream kinase tyrosine‐protein kinase ITK/TSK (ITK), thus directly influencing T‐cell function [116, 117]. In this line, a study using scRNA‐seq found that T‐cell subtypes respond to ibrutinib therapy with shared transcriptional changes, including downregulation of genes involved in leukocyte function and cell–cell interactions, as well as upregulation of genes involved in quiescence and senescence [26].

Similarly, another study reported that, in patients with monoclonal B‐cell lymphocytosis (MBL)/CLL, interactions—including inhibitory T‐cell signals like BTLA/MIF–TNFRSF14, CTLA4–CD86, and LGALS9–HAVCR2—were downregulated after ibrutinib treatment [27].

The consequences of these quiescent and less inhibited T‐cell states after ibrutinib treatment remain to be further investigated. Although it has been suggested that the quiescent state would render T cells less fit and could be linked to a higher susceptibility to infections [26], a lower inhibition of T cells could improve anti‐tumor T‐cell responses.

Studies focusing on the mechanisms driving treatment response found that, in MCL, the proportion of CD8 T_EF_ cells in the peripheral blood (PB) of ibrutinib responders was higher compared to non‐responders. However, ibrutinib resistance was associated with an increased gene expression of ligand–receptor pairs such as *LGALS1*–*CXCR4*/*CD69* and *TGFB1*–*CXCR4*, suggesting that T‐cell suppression likely plays a role in therapeutic resistance [25]. In CLL, spectral flow cytometry analysis of PB from 10 ibrutinib‐responsive and eight relapsing patients showed an increase of proliferating CD8 T cells as well as in CD4 T_FH_ cells in relapsed patients [28].

#### PD‐1 blockade

Responses to anti‐PD‐1/PD‐L1 treatment in B‐cell lymphomas vary considerably, with generally disappointing outcomes [118]. The aggressive transformation of a CLL, Richter syndrome (RS), has shown a significant 43–65% response rate [119, 120]. Parry *et al*. [24] delved into the mechanisms determining response to PD‐1 therapy in RS using scRNA‐seq. The authors found that the treatment response was linked to increased levels of clonally expanded CD8 T_EF/EM_ cells. Instead of resembling Tpex cells, these cells were inferred to differentiate away from terminally exhausted CD8 T cells and had high expression of tissue‐resident T‐cell transcription regulator protein ZNF683, which regulates pathways of T‐cell activation and cytotoxicity [24].

Because of the mediocre results of single‐agent treatments, combination therapies of kinase inhibitors and checkpoint blockade are being investigated [121]. However, according to the current clinical trial results, the clinical benefit of PD‐L1 blockade in combination with ibrutinib in DLBCL or relapsed/refractory FL patients is not obvious [122]. As previously mentioned, the use of other immune checkpoint blockades instead of PD‐1 might be more effective in B‐NHL, given the high abundance of inhibitory molecules such as CD39 (*ENTPD1*), LAG‐3, TIM‐3 (*HAVCR2*) and TIGIT.

### CAR‐T

CAR‐T cell therapy has transformed the treatment landscape for hematological malignancies, with real curative results for some patients with relapsed or refractory disease [123]. Despite its success, CAR‐T cell therapy still faces some challenges, including resistance, relapse, and adverse events. Comprehensive reviews on the current progress and challenges of CAR‐T therapy in B‐NHL already exist [124, 125, 126]. Here, insights gained with single‐cell technologies to delve deeper into the molecular and cellular mechanisms driving treatment response are summarized.

#### CAR‐T cell dynamics after infusion

CAR‐T cells follow multiphasic kinetics after infusion, with an initial expansion and differentiation phase, followed by contraction and persistence. The dynamics of these phases are determined by aspects such as the heterogeneity of the infusion product (IP) and the host TME. ScRNA‐seq combined with T‐cell receptor beta repertoire examination revealed that clonal diversity was highest in the IP and decreased after infusion in B‐NHL patients [41]. In addition, CAR‐T cells acquired a more activated and cytotoxic transcriptional profile early after infusion [42], which again declined at later time‐points, when genes associated with a ‘resting’ phenotype increased, alongside tumor clearance [41]. Interestingly, a 10‐year follow‐up of two CLL patients who achieved complete response revealed that, after the initial CD8 T‐cell‐dominated response, the long‐term remission stage was prevailed by proliferative and cytotoxic CD4 CAR‐T cells [38].

#### Deciphering the efficacy of CAR‐T therapy

Because of the heterogeneous nature of the CAR‐T cell products, multiple factors contribute to treatment efficacy. Extrinsic factors include tumor load and the amount of antigen it expresses [127, 128]. Intrinsic ones are related to the CAR construct structure, including the co‐stimulatory domain, the CD4/CD8 ratio, and the initial phenotypic status of the T cells [129]. Intensive investigation has focused on identifying predictive biomarkers of response.

On the one hand, several single‐cell studies report an association between response and a higher frequency of CAR‐T cells with a memory or stem‐like phenotype in both the initial infusion product and after expansion in PB in DLBCL, MCL, and CLL [29, 31, 35, 40, 44]. Of note, some discrepancies exist between studies. For example, analyzing the CAR‐T products 7 days post‐infusion of 31 DLBCL patients using CyTOF, Good *et al*. [34] found that, instead of the memory subset, the effector CD4 and CD8 CAR‐T cells with senescence features were increased in patients who experienced durable disease control. In addition, differences in dynamics between CAR‐T designs, i.e. axicabtagene ciloleucel (axi‐cel) and tisagenlecleucel (tisa‐cel), have been observed using scRNA‐seq analyses: whereas expansion of proliferative CD8 T_EM_ CAR‐T was observed in DLBCL patients treated with tisa‐cel, more heterogeneous populations were predominant in responders treated with axi‐cel [30, 31].

On the other hand, it has been consistently reported by multiple groups using both protein‐ and RNA‐based single‐cell analyses that patients who did not respond had higher frequencies of both CD4 and CD8 T_EX_ cells [29, 35, 39], as well as higher frequencies of CD4 T_REG_ cells [31, 34]. Determinants of relapse to CAR‐T cell therapy in MCL and DLBCL also involve an acquisition of an exhaustion phenotype, with CAR‐T cells showing increased expression of inhibitory receptors as well as an impaired cytotoxic function, as determined by scRNA‐seq and imaging mass cytometry [36, 37]. As a result of these observations, combination of CAR‐T cell therapy with IC blockage has been suggested. In this line, Jackson *et al*. [42] reported that TIGIT blockade improved the anti‐tumor function of CAR‐T cells *in vivo*.

#### Adverse events

Additional lines of research currently focus on identifying the cellular and molecular mechanisms driving severe side effects, such as prolonged cytopenia, cytokine release syndrome (CRS), and immune effector cell‐associated neurotoxicity syndrome (ICANS). Prolonged cytopenia is defined as grade ≥ 3 reduction in the number of mature blood cells that lasts longer than 30 days after treatment initiation [130]. It occurs in approximately 30% of CAR‐T‐treated patients, and it has significant clinical implications, including an increased risk of infection [131]. Interestingly, Strati *et al*. [33] analyzed 14 BM samples of CAR‐T‐treated refractory/relapsed LBCL patients *via* scRNA‐seq and found that a subset of CD8 T cells expressing CX3C chemokine receptor 1 (CX3CR1) and INF‐γ were significantly enriched in patients with prolonged cytopenia compared to those without. CRS is caused by a large and rapid release of cytokines into the blood, which induces systemic inflammation [131]. CRS is considered a cofactor or initiating event of ICANS, which usually manifests as a toxic encephalopathy as a result of blood–brain barrier permeability and subsequent infiltration of proteins, T cells, and cytokines, causing neuronal cell injury [132, 133]. Single‐cell multiplex cytokine profiling on the IPs showed that a higher polyfunctional strength index (PSI) in the CAR‐T cell product is associated with clinical response but also severe CRS [43]. The same study also identified IL‐17A‐polyfunctional CAR‐T cells to be associated with severe ICANS. In contrast, a higher frequency of T_REG_ cells, which is associated with a dismal outcome, was linked to less severe neurotoxicity [34].

## Conclusions and perspectives

Recent analyses with single‐cell technologies have been central to advancing our understanding of the B‐NHL microenvironment. The T‐cell landscape has been refined to unprecedented levels, and the biological and clinical relevance of each cell population or state in the TME is starting to be understood. However, these technologies also pose unique challenges that need to be addressed to efficiently increment and translate this knowledge into the clinic.

First, a critical challenge lies in the differences between protein‐ and RNA‐based single‐cell techniques. Multiparameter flow cytometry robustly detects protein markers, such as those defining T_H_ subsets, whereas scRNA‐seq often misses these as DEGs, potentially overlooking functionally relevant subsets. Critically, it would be inaccurate to assume that transcriptional profiles obtained by scRNA‐seq define the functional state of a cell subset. This is particularly relevant in processes tightly regulated at the posttranscriptional level, such as cell signaling (e.g., JAK–STAT [134] or NF‐κB pathways [135]) and metabolism (e.g., glycolysis or oxidative phosphorylation [136]), where mechanisms such as microRNA regulation, protein degradation, or translation efficiency significantly contribute to mRNA‐protein discordance. In contrast, transcriptomics and epigenomics uniquely reveal a cell's molecular potential through accessible chromatin or mRNA expression, enabling prediction of functional states before protein‐level changes. Thus, generating optimally designed experiments that integrate transcriptomic, proteomic, and functional assays will be key for a comprehensive T‐cell profiling.

Second, there is an imperative need for consensus on cell type and state nomenclature in order to allow for cross‐study comparison. Initiatives like the Human Cell Atlas currently aim to tackle this issue by using new computational approaches leveraging artificial intelligence (AI) to construct human cell atlases in health and disease settings [137].

Third, due to their high cost, most studies have involved relatively small cohorts, limiting the statistical power of the analyses and thus reducing the identification of clinically relevant factors.

Fourth, and as part of a solution to the previous challenge, the integration of single‐cell datasets needs a flexible but rigorous statistical and computational framework that is only now starting to be developed [138]. Such standardization is crucial to enable a significant increase in sample size to generalize and corroborate findings.

Last, the data generated by single‐cell studies is largely descriptive and mostly hypothesis‐generating, necessitating additional functional studies to validate many of the findings. Thus, extracting the relevant information and generating optimally designed experiments will be key for transforming single‐cell data into relevant biological knowledge.

Despite these challenges, single‐cell technologies prompt many new opportunities. For instance, we can now link the transcriptional state to the TCR repertoire, which provides key information of antigen specificity and response, as well as longitudinal clonal dynamics and heterogeneity of the T‐cell response. Besides, numerous techniques have been developed to identify tumor‐reactive T cells [138, 139, 140, 141, 142], the understanding of which will eventually impact the design and application of T‐cell therapies. Next, deconvolution methods such as CIBERTSORTx [143], DeconRNAseq [144], dtangle [145], and MuSiC [146] have been developed to infer the cellular composition in bulk RNA data. These methods render large bulk data useful to validate observations from single‐cell studies, enhancing their statistical robustness. Additionally, using multi‐omics approaches to study T cells will significantly advance our understanding of the molecular mechanisms driving specific T‐cell functions in B‐cell neoplasms. Last, integration of single‐cell genomics, epigenomics, transcriptomics, proteomics, metabolic, and spatial information will enable simultaneous target discovery, validation, and searching for upstream and downstream molecules and pathways. The use of AI in multi‐omics data of large‐scale clinical cohorts will help determine risk prediction, prognosis, therapeutic classification, and personalized therapy. Altogether, these innovations are expected to pave the way for improved patient stratification and novel therapeutic strategies in B‐NHL.

## Author contributions

LL‐C revised the literature and wrote the manuscript.
